# Novel echocardiographic classification of mitral valve structure in atrial septal defect of adult and its value on surgical repair for mitral valve

**DOI:** 10.3389/fmed.2026.1861780

**Published:** 2026-08-12

**Authors:** Yuxi Li, Lin Zhao, Xuezeng Xu, Liang Cao, Guomeng Jiang, Jianlong Yang, Wei Bai, Chennian Xu, Liwen Liu, Xin Meng

**Affiliations:** 1 Department of Ultrasound, Xijing Hospital, The Fourth Military Medical University, Xi’an, Shaanxi, China; 2 Department of Cardiovascular Surgery, Xijing Hospital, The Fourth Military Medical University, Xi’an, Shaanxi, China

**Keywords:** atrial septal defect, mitral regurgitation, mitral valve, three-dimensional echocardiography, transesophageal echocardiography

## Abstract

**Objectives:**

To evaluate the differences in mitral valve (MV) structure in adult patients with atrial septal defect (ASD) by transesophageal echocardiography (TEE) and explore the clinical value of these differences in surgical repair for mitral valves.

**Methods:**

The data related to the mitral valve from 75 adult patients who underwent surgical repair were collected which formed the observation group. The corresponding data from 63 adult patients with normal intracardiac structures and minimal or no mitral regurgitation were collected. The structural differences of the mitral valve between the two groups compared, and ASD-MV based on the degree of these differences were classified.

**Results:**

There were significant differences in the morphology and structure of the mitral valve between the observation group and the control group. Statistical analysis revealed significant differences in ASD area, Qp/Qs ratio(the ratio of pulmonary to systemic blood flow), and mitral valve anterior leaflet length across ASD-MV subtypes (all *P* < 0.05).

**Conclusion:**

This study classified the mitral valve in patients with ASD to assist in determining whether concurrent mitral valve repair is indicated. It significantly reduced the proportion of patients with markedly aggravated mitral regurgitation post-ASD repair, providing a reference for concurrent mitral valve intervention in ASD patients.

## Introduction

Atrial septal defect (ASD) refers to an abnormality in the development of the atrial septum during embryonic growth, resulting in an unsealed defect between the left and right atria. This condition accounts for approximately 10% of all congenital heart diseases and 20–30% of adult congenital heart diseases (1). Studies indicate that about 10–20% of ASD patients experience worsening mitral regurgitation (MR) after transcatheter atrial septal defect occlusion (2). Approximately 29–35% of patients exhibit aggravated mitral regurgitation post-surgical ASD closure (3). With the continuous development of surgical techniques and imaging technologies, minimally invasive treatment for ASD has become feasible. However, there are still reports of aggravation of mitral regurgitation after different treatments (4–8). Therefore, careful surgical planning is necessary for patients with ASD combined with mitral valve disease. A thorough understanding of the anatomical structure and hemodynamics of such patients is crucial for determining the treatment approach and prognosis (9). However, although there have been a certain number of reports on ultrasound imaging studies for such patients (10–13), there may be a lack of targeted ultrasound imaging research reports for different treatment methods.

Clinically, some patients develop mitral regurgitation immediately after discontinuing extracorporeal circulation or during follow-up, ultimately requiring re-operation. Secondary surgery not only increases surgical risks but also imposes additional financial burdens. Therefore, timely detection of mitral valve structural abnormalities before ASD repair and concurrent surgical intervention are crucial for reducing re-operation rates. Previous studies have explored risk factors for predicting preoperative worsening of mitral regurgitation, while research on the structural differences between adult ASD patients and normal adults remains limited (2, 14, 15). Previous studies on secondary ASD are mostly limited to small samples or case reports, and large-scale supportive studies are still insufficient, which is the key clinical issue that the present study aims to supplement. Whether these differences influence the timing and approach of mitral valve repair still requires further investigation. The objective of this study is to evaluate mitral valve structural characteristics in adult ASD patients and classify them based on morphological features, thereby providing theoretical guidance for mitral valve repair procedures.

## Materials and methods

### Data and protocal

A total of 75 adult patients who underwent total thoracoscopic ASD repair surgery at Xijing Hospital from March 2022 to September 2023 were enrolled in the observation group. The inclusion criteria were as follows: (1) A secondary ASD that was not suitable for ASD closure, that the ASD is too large in size or its location is unsuitable for occluder implantation.; (2) Age > 18 years; (3) No contraindications to transesophageal echocardiography (TEE).

The exclusion criteria were as follows: (1) Arrhythmia; (2) Coexisting structural or functional cardiac diseases other than tricuspid regurgitation and ASD, such as cardiomyopathy or rheumatic heart disease; (3) History of cardiac surgery, including pacemaker implantation; (4) Severe hepatic or renal dysfunction; (5) Incomplete clinical data.

The control group comprised 63 patients who underwent TEE for screening of patent foramen ovale (PFO) due to dizziness or headache. All subjects had normal cardiac structure. They were enrolled to serve as a reference for mitral valve morphological analysis. The inclusion criteria were as follows: (1) Normal cardiac structure, no or minimal regurgitation in all valve groups, and PFO shunt width < 1 mm; (2) Age > 18 years; (3) No TEE contraindications.

The exclusion criteria included the same as items (1), (3), and (4) in the observation group, plus patients with underlying conditions affecting cardiac structure.

The aforementioned research protocol has been approved by the Ethics Committee of Xijing Hospital, The Fourth Military Medical University (No.KY20252234-C-1).

The baseline characteristics of the patients were as follows: The observation group comprised 75 patients aged 18–59 years, including 46 females (61.3%) and 29 males (38.7%); the control group consisted of 63 patients aged 20–62 years, including 37 females (58.7%) and 26 males (41.3%) (Table 1).

**TABLE 1:** Comparison of general data of two groups.

Group	*n*	Gender* (Female/Male, *n*)	Age	BMI (kg/m^2^)	Heart rate (beats/min)	hypertension* (*n*%)	diabetes mellitus* (*n*%)
OG	75	46/29	38.9 ± 14.0	23.2 ± 1.8	87.3 ± 10.7	8(10.6%)	4(5.3%)
CG	63	37/26	40.1 ± 16.3	23.7 ± 1.6	77.8 ± 9.1	8(12.7%)	3(4.8%)
*t*	–	–	1.191	1.321	3.725	–	–
*P*	–	0.383	0.451	0.672	0.013	2.315	1.734

–: No relevant data. * Fisher’s exact test.

### Equipment

The system consists of a GE Vivid E9 (General Electric Company, Boston, United States) or Philips EPIQ 7C color Doppler ultrasound diagnostic system (Philips Company, Eindhoven, Netherlands), both equipped with a three-dimensional (3D) transesophageal probe (6VT of GE and X8-2T of Philips) and 3D image in-machine analysis software (Flexi-Slice of GE and MultiVue of Philips).

### Data collection and measurement methods

All patients in the observation group underwent TEE to obtain preoperative cardiac ultrasound data. Anesthesia was induced with etomidate combined with short-acting analgesics while maintaining spontaneous breathing. The anesthetic state remained stable with minimal impact on circulation, fully meeting the requirements for TEE examination.

**FIGURE 1:**
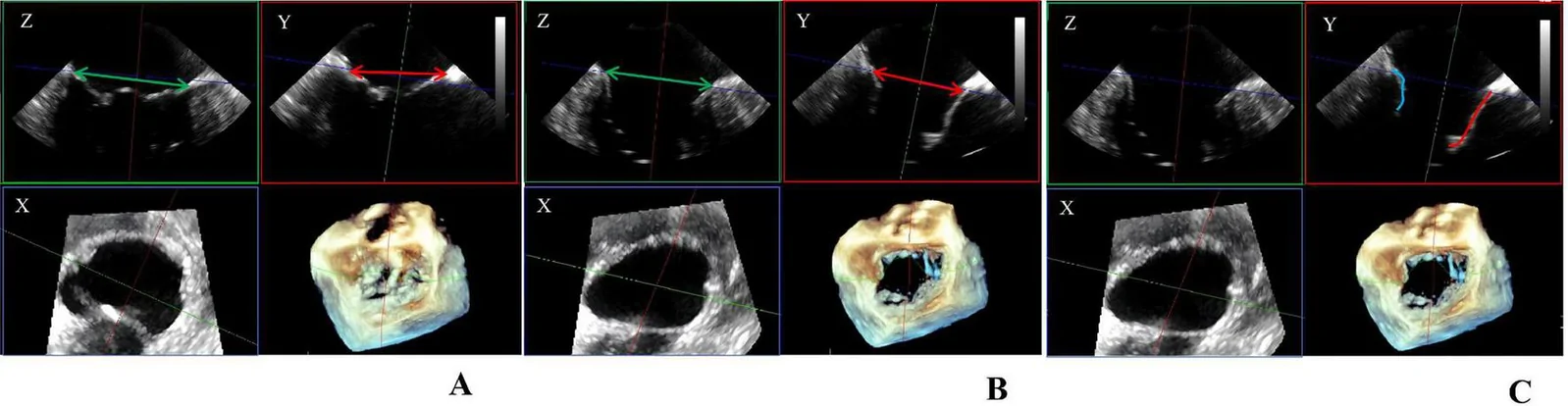
Manual segmentation of dynamic mitral valve bird’s-eye view using three-dimensional processing software. **(A)** The systolic mitral annular commissural diameter (green line) and anteroposterior diameter (red line). **(B)** The diastole mitral annular commissural diameter (green line) and anteroposterior diameter (red line). **(C)**: The lengths of the anterior leaflet (red line) and posterior leaflet (blue line) in segment 2 during diastole. A2: segment A2 of anterior mitral leaflet; P2: segment P2 of posterior mitral leaflet.

Acquire 2D images of the mitral valve covering 3–4 cardiac cycles as well as three-dimensional surgical en face view (3D-Zoom) of the mitral valve with a volume frame rate of ≥ 20 Vol/s, and measure its structural parameters at two phases: late diastole and late systole (Figure 1). Key parameters included: annular diameter (anterior-posterior and commissural), leaflet length (anterior and posterior) (Figure 1), Mitral valve coaptation depth (also referred to as tent height, defined as the perpendicular distance from the coaptation point to the mitral annulus plane) and mitral valve coaptation length (the overlapping length between the two mitral leaflets at their coaptation zone). The total leaflet length/annulus dimensions(anterior-posterior and commissural), maximum forward flow velocity across the mitral valve, and the ratio of pulmonary to systemic blood flow (Qp/Qs). The specific measurement method is shown in Figure 1. Manual segmentation of dynamic mitral valve bird’s-eye view using three-dimensional processing software. The X-plane passes through the attachment points of both A2 and P2 leaflets simultaneously (equivalent to the virtual mitral annular plane at the highest point of the mitral annulus). The Y- and Z-planes are both perpendicular to the X-plane; the Y-plane traverses the midpoint connecting lines of the A2 and P2 annuli, while the Z-plane runs along the maximum left-right diameter of the annulus. On the Y-plane, measure the mitral annular anteroposterior diameter, lengths of the anterior and posterior leaflets in segment 2, coaptation length and coaptation depth. On the Z-plane, measure the mitral annular commissural diameter.

The control group underwent TEE under topical anesthesia, and corresponding mitral valve data were collected for comparison. For the observation group, the four-chamber view, right ventricular inflow/outflow tract view and biatrial view were additionally obtained to measure the diameter of atrial septal defects, and the elliptical area formula was adopted to calculate the defect area (This method was used because the ASD in most patients of the observation group were too large to be fully visualized via three-dimensional imaging).

**FIGURE 2:**
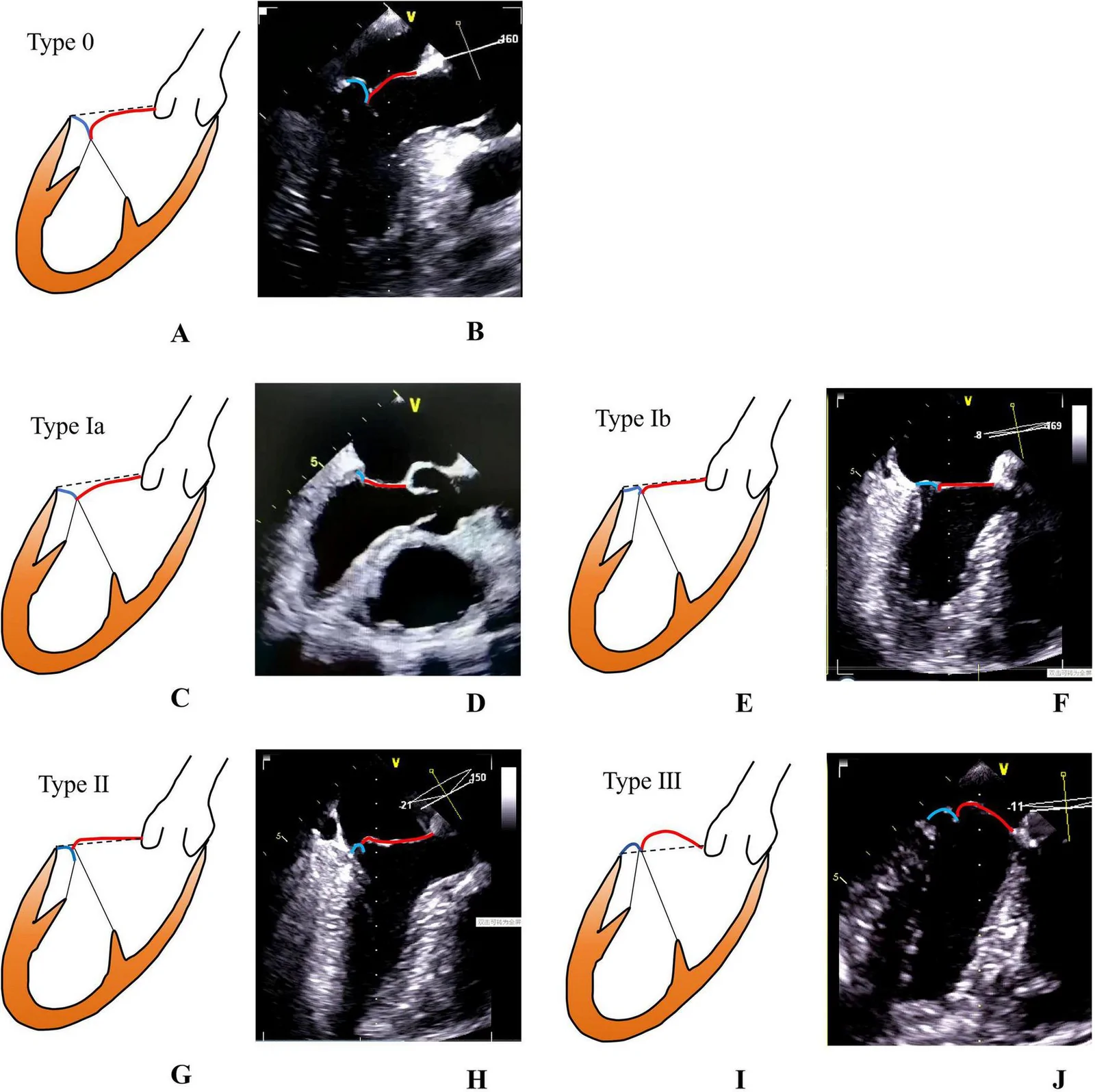
TEE and Schematic diagram of ASD-MV classification. **(A,C,E,G,I)** The schematic diagrams which black dashed line represents the annular plane. **(B,D,F,H,J)** Mitral valve transesophageal echocardiogram matching the schematic illustration on the left (The red line represents the anterior leaflet and the blue line represents the posterior leaflet).

Two senior echocardiographers independently reviewed all images in a double-blind manner, completed measurements of study data, and determined the classification of atrial septal defect-mitral valve (Figure 2), with good consistency in their diagnostic results.

### Intraoperative and postoperative follow-up

Mitral valve repair shall be performed concurrently on patients falling into the following three categories: 1. Patients with moderate or greater mitral regurgitation; 2. Patients with significantly abnormal mitral valve morphology (ASD-MV Type II and Type III) accompanied by mitral regurgitation; 3. Patients in whom TEE detects MR increased by two grades or more immediately after ASD repair. Details are as follows: Thirty-three patients underwent mitral valve repair during the same procedure for ASD repair, with the following clinical features: twenty-six cases had significant mitral valve malformations (Figures 2G–J, ASD-MV Type IIV T Type III); four cases presented moderate or severe preoperative mitral regurgitation; and three cases developed increased mitral regurgitation (above moderate level) immediately after cardiopulmonary bypass termination.

For cases where the valve tips exceeded the annular plane, Gore-Tex sutures were used to reconstruct artificial chordae tendineae, restoring the valve tips to a relatively normal position. Then, the original mitral annulus was reduced using an artificial mitral annulus, increasing the Coaptation length and depth to minimize MR. In cases where the valve tips did not exceed the annular plane, only the annulus was reduced using an artificial mitral annulus. After surgery, the coaptation morphology of mitral leaflets was significantly improved, with coaptation depth and coaptation length increased by 2–5 millimeters; regurgitation disappeared immediately or only trace mitral regurgitation remained.

The observation group was examined by TTE at discharge, 6 months, and 1 year after the operation to observe the degree of MR, the velocity of transmitral flow, and the changes in the cardiac cavity.

The severity of MR was assessed using a guideline-based multiparametric echocardiographic method. Quantitative parameters including vena contracta width, proximal isovelocity surface area (PISA), effective regurgitant orifice area (EROA) and regurgitant volume were combined with conventional color Doppler imaging for comprehensive evaluation. MR was categorized as trivial, mild, moderate or severe. MR progression was defined as an increase of ≥ 2 severity grades compared with the preoperative baseline at any follow-up time point. All echocardiographic assessments were performed by experienced sonographers.

### Statistical methods

Statistical analysis was performed using SPSS 27.0 software (IBM Corporation, Armonk, New York, United States). The normality of the data was assessed using the K-S test. Quantitative data conforming to a normal distribution were presented as means ± standard deviations (**$\bar{\text{x}}$** ± s). An independent samples *t*-test was employed for inter-group comparisons.

Univariate analysis of variance (ANOVA) was used to compare measurement data from different time points (≥3) in the same patient. The LSD-t method was applied for statistically significant comparisons between groups at different time points.

Spearman’s test was utilized to analyze the correlation between ordered but non-normal data. Categorical variables were expressed as case counts (percentage) [n (%)], and Fisher’s exact test was used for comparison. The *P* < 0.05 was considered statistically significant.

## Results

### Morphological characteristics of the mitral valve on preoperative TEE imaging in the observation group

Compared with the control group, only 16 cases (21.3%) in the observation group exhibited normal mitral valve morphology, while the remaining 59 cases (78.7%) showed varying degrees of morphological abnormalities. Mitral valves in patients with ASD were classified according to the position of mitral leaflet tips and leaflet bodies (above or below the annular plane) and coaptation length, and this classification was designated the adult ASD-MV classification. These were classified into four types (Figure 2).

Type 0 (16 cases, 21.3%): The mitral valve has a normal morphology. Both the valve leaflet tips and the leaflet body are located below the annular plane, and the coaptation length ≥ 3 mm (Figures 2A,B).

Type I: The valve leaflet tips are located below the annular plane, with the coaptation length < 3 mm. Based on whether the valve leaflet body is below the annular plane, it is further divided into Type Ia (12 cases, 16.0%, with the valve leaflet body below the annular plane, Figures 2C,D) and Type Ib (19 cases, 25.3%, with the valve leaflet body at or above the annular plane, and no coaptation depth, Figures 2E,F).

Type II (19 cases, 25.3%): The anterior valve leaflet body is bulging, with the leaflet tips at or above the annular plane, overlapping the posterior leaflet body and causing misalignment of the closure point (Figures 2G,H).

Type III (9 cases, 12.0%): Both the anterior and posterior leaflet bodies are bulging, with the leaflet tips at or above the annular plane (Figures 2I,J).

### Comparison of mitral valve structure and other parameters among different subtypes in the observation group

Comparison of mitral valve structural parameters among various ASD-MV subtypes (including anteroposterior and commissural diameters of the annulus, leaflet length, total leaflet length/anteroposterior diameter, total leaflet length/commissural diameter, and annulus anteroposterior diameter/commissural diameter), ASD area, and analysis of the Qp/Qs value differences and their correlation with ASD area.

The results suggest that there is a significant positive correlation between ASD area and Qp/Qs value (correlation coefficient *r* = 0.634, *P* < 0.05). The ASD area in the 0-type group was significantly smaller than the mean values of the II and III groups (*P* < 0.05), and the Qp/Qs value was significantly lower than the mean values of the Ib, II, and III groups (*P* < 0.05), indicating that larger ASD areas or higher Qp/Qs values are associated with a greater likelihood of significant morphological abnormalities in the mitral valve.

**FIGURE 3:**
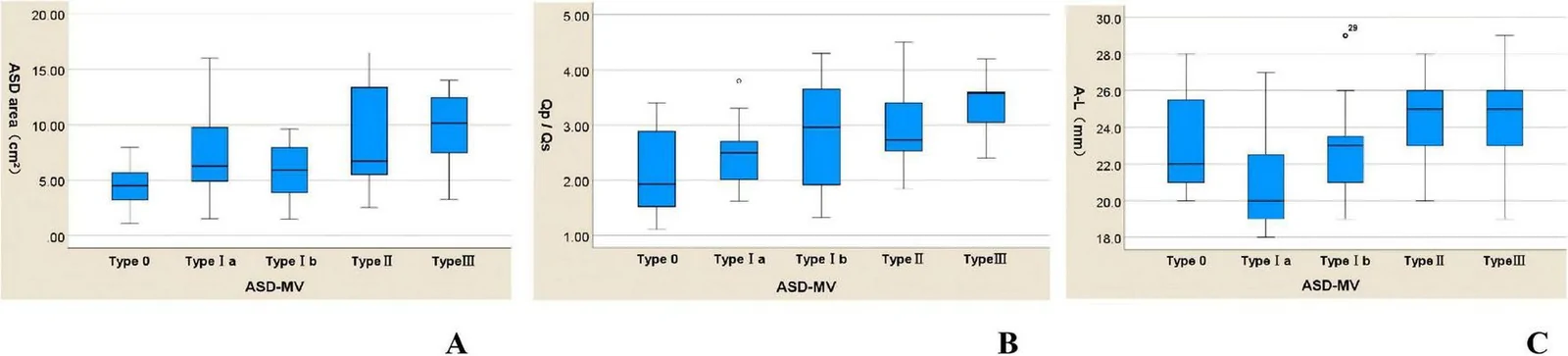
Comparison of mitral valve structure and other parameters among subtypes in the observation group. **(A)** ASD area; **(B)** Qp/Qs; **(C)** A-L. Note: ASD area, area of atrial septal defect; Qp/Qs, ratio of pulmonary to systemic blood flow; A-L, anterior mitral leaflet length.

The anterior leaflet length of the mitral valve in the II and III groups was longer than that in the 0-type group (*P* < 0.05), which may be attributed to the more pronounced leftward displacement of the ventricular septum caused by larger ASDs, leading to mitral valve prolapse. The anterior leaflet may compensate for mitral regurgitation by increasing in length. In contrast, the anterior leaflet of the Ia group was shorter than that of the 0-type group (*P* < 0.05) (Figure 3).

No statistically significant differences were observed in other mitral valve parameters among the subtypes.

### Comparison of MV and other parameters between the observation group and control group

The observation group exhibited smaller commissural mitral annular diameters, reduced mitral valve coaptation depth, shorter coaptation length, shorter posterior leaflet length, and lower maximum transmitral flow velocity compared to the control group (all *P* < 0.05). Both systolic and diastolic mitral annular anteroposterior diameters were smaller in the observation group than in the control group, with the systolic difference being statistically significant (*P* < 0.05). Among the observation group, 63 cases (84.0%) had posterior leaflet lengths ≤ 10 mm, compared to only four cases (6.3%) in the control group. The total leaflet length/annular anteroposterior diameter and total leaflet length/annular left-right diameter were both smaller in the observation group than in the control group (*P* < 0.05). The pulmonary/ systemic circulation blood flow ratio (Qp/Qs) was significantly higher in the observation group (*P* < 0.05). Both systolic and diastolic left ventricular anteroposterior diameters were smaller in the observation group than in the control group (*P* < 0.05), as detailed in Table 2.

**TABLE 2:** Comparison of left ventricular size and mitral valve (MV) related parameters between the two groups ($\bar{\text{x}}$ ± s).

LV and MV-related parameters	Observation group (*n* = 75)	Control group (*n* = 63)	*F*	*P*
LVEDD (mm)	42.55 ± 3.79	45.97 ± 3.56	0.034	<0.001
LVESD (mm)	29.25 ± 3.38	31.75 ± 3.91	0.971	<0.001
CD (S) (mm)	32.54 ± 3.18	34.80 ± 2.89	0.113	0.020
CD (D) (mm)	31.84 ± 3.13	33.68 ± 3.03	0.021	0.003
A-PD (S) (mm)	26.35 ± 2.69	27.93 ± 2.70	1.168	<0.001
A-PD (D) (mm)	26.53 ± 2.38	27.23 ± 2.62	1.376	0.170
A-L (mm)	23.22 ± 2.76	23.70 ± 2.30	3.023	0.280
P-L (mm)	8.88 ± 1.75	13.23 ± 2.27	1.305	<0.001
CPD (mm)	1.74 ± 1.96	4.38 ± 1.78	0.092	<0.001
CPL (mm)	2.14 ± 1.05	4.51 ± 0.99	0.882	<0.001
A + P/CD (S)	0.98 ± 0.16	1.07 ± 0.12	0.883	<0.001
A + P/A-PD (S)	1.21 ± 0.19	1.33 ± 0.14	0.195	<0.001
A-PD/CD (S)	0.81 ± 0.7	0.80 ± 0.06	0.029	0.249
Qp/Qs	2.81 ± 1.15	1.12 ± 0.74	1.048	<0.001
MV-Vmax (cm/s)	70.34 ± 30.51	81.28 ± 26.87	1.473	0.017

LVEDD, left ventricular diastolic diameter; LVESD, left ventricular systolic diameter; CD (S), commissural diameter systole; A-PD (S), anterior-posterior diameter systole; CD (D), commissural diameter diastole; A-P D (D), anterior-posterior diameter diastole; A-L, anterior leaflet length; P-L, posterior leaflet length; CPD, Coaptation depth; CPL, Coaptation length; A + P/CD (S) total leaflet length/commissural diameter systole; A + P/A-PD (S), total leaflet length/anterior-posterior diameter systole; A-PD/CD (S), anterior-posterior diameter systole/commissural diameter systole; Qp/Qs, Pulmonary-to-Systemic Flow Ratio; MV-Vmax, aximum trans-mitral valve flow velocity.

### Preoperative and immediate postoperative regurgitation in patients undergoing mitral valve repair during the same period in different subtypes of adult ASD-MV

All 75 patients in the observation group successfully underwent atrial septal defect repair, with 33 cases undergoing concurrent mitral valve repair. The specific cases are reported in detail as follows.

A case of MV repair with Type 0 valve. Due to moderate MR preoperatively, a mitral valve annuloplasty was performed using an artificial annuloplasty ring.

A total of 3 cases of MV repair Type Ia, Among them, 2 cases had moderate MR preoperatively, and 1 case developed immediate postoperative regurgitation that progressed from mild to moderate. All 3 cases underwent annuloplasty using an artificial annuloplasty ring.

A total of 4 cases of MV repair Type Ib, Among them, 2 cases had moderate MR preoperatively, and 2 cases developed immediate postoperative regurgitation that progressed from mild to moderate, using the same repair method as Type Ia.

There are 19 patients with Type II. This type exhibited significant abnormalities in the anterior leaflet morphology. Among these, 16 cases with preoperative mitral regurgitation underwent MV repair (3 cases without regurgitation were left untreated). The procedure involved first using Gore - Tex sutures to reconstruct the valve cusps to a relatively normal position, followed by annuloplasty using an artificial annuloplasty ring.

There are nine patients with Type III. This type exhibited significant abnormalities in both the anterior and posterior leaflets, along with mitral regurgitation. All underwent mitral valve repair, which involved first using artificial sutures to reposition the anterior and posterior leaflet cusps, followed by annuloplasty using an artificial annuloplasty ring.

Distribution of mitral regurgitation in different ASD-MV classifications as detailed in Table 3.

**TABLE 3:** Distribution of mitral regurgitation severity across different ASD-MV classifications (preoperative).

MR classification	No-MR (n)	Trivial (*n*)	Mild (*n*)	Moderate (*n*)	Severe (*n*)	MV repair (*n*)
Type 0 (*n* = 16)	7	5	3	1	/	1
Type Ia (*n* = 12)	3	7	2	2	/	3
Type Ib (*n* = 19)	4	11	2	2	/	4
Type II (*n* = 19)	3	7	8	4	/	16
Type III (*n* = 9)	/	2	4	3	/	9

**FIGURE 4:**
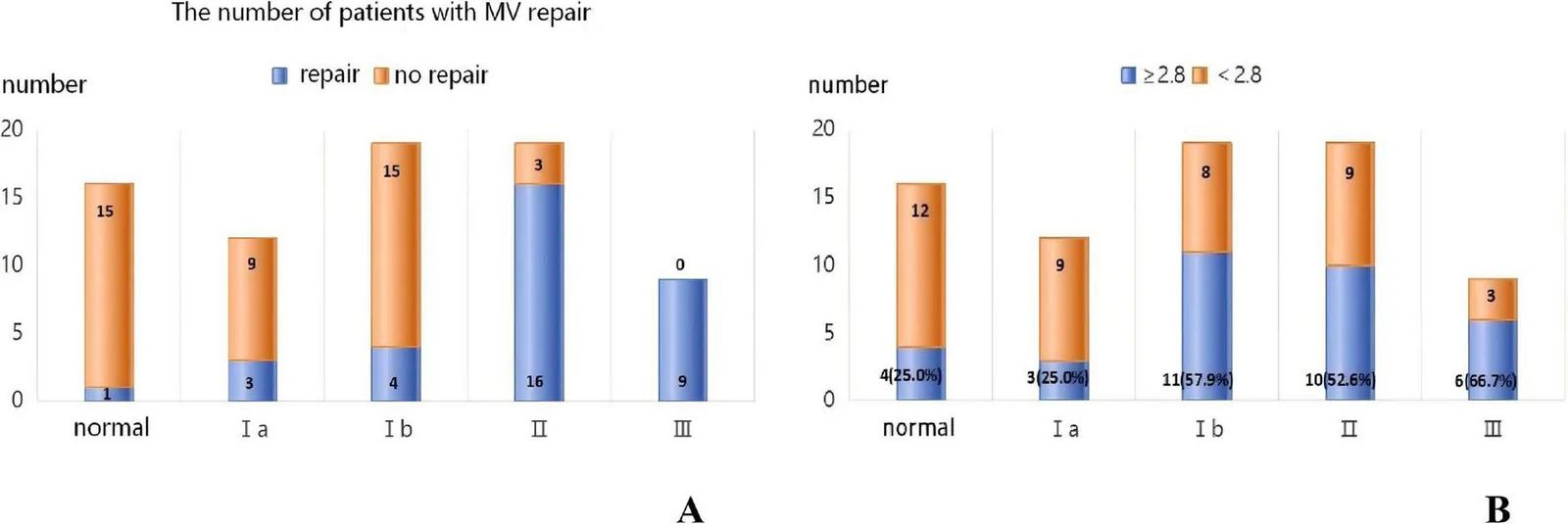
Distribution of the number of patients with MV repair in different subtypes of ASD-MV and distribution of patients with Qp/Qs ≥ 2.8 in different classifications of the mitral valve. **(A)** The proportion of patients undergoing mitral valve repair in the same period among various subtypes. **(B)** The proportion of patients with Qp/Q ≥ 2.8 in each subtype.

**FIGURE 5:**
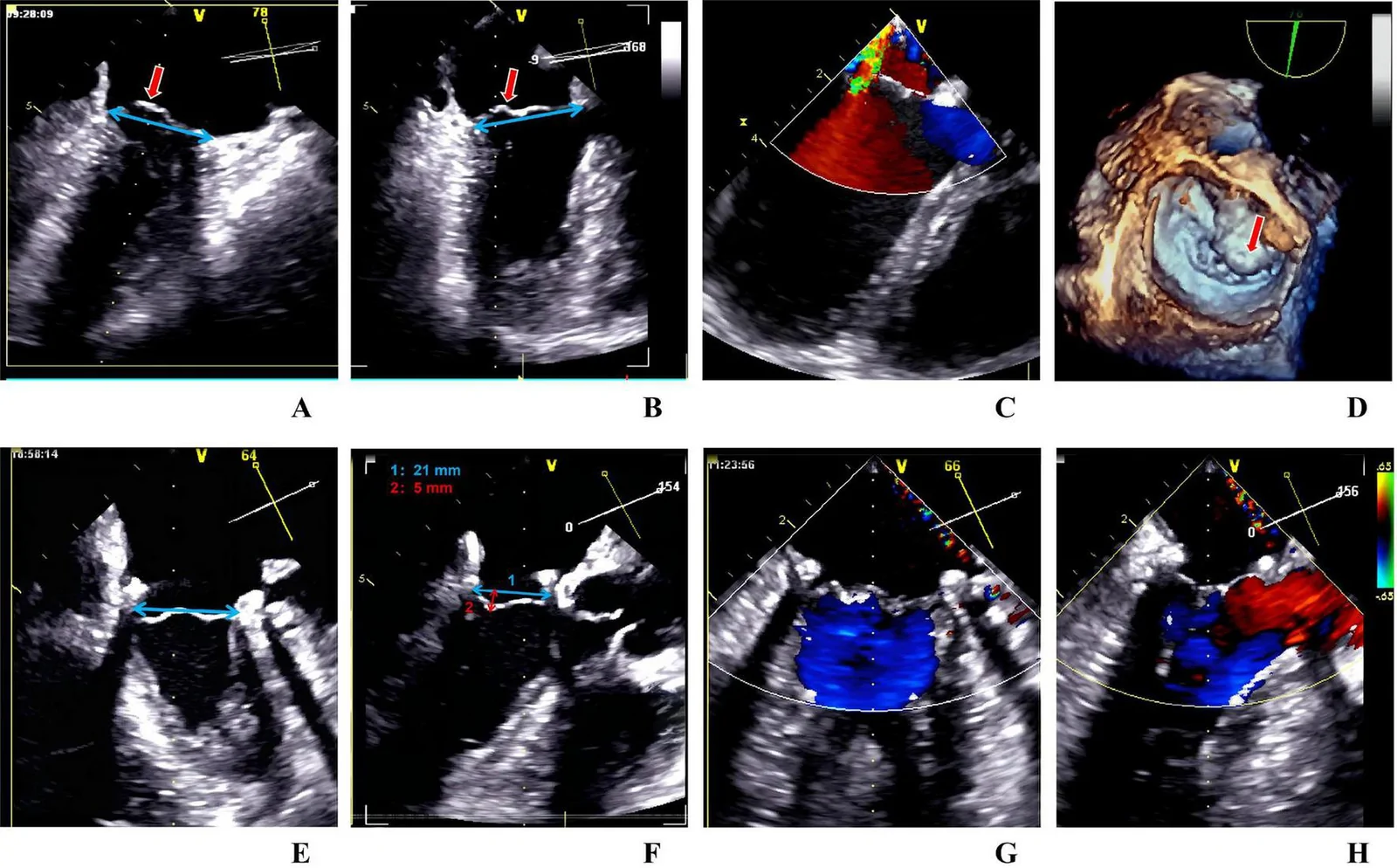
Preoperative TEE results of ASD-MV type III patients and TEE results immediately after MV repair surgery. **(A)** Mitral commissural view shows bulging of the anterior mitral leaflet into the left atrium. **(B)** The mitral valve long-axis view demonstrates that both the anterior and posterior leaflets bulge into the left atrium beyond the annular plane. **(C)** Minor mitral regurgitation. **(D)** The three-dimensional overview of the MV intuitively demonstrates significant protrusion of the anterior leaflet. **(E)** MV leaflets return below the annular plane after repair. **(F)** The coaptation depth increased to 5 mm which was showed in red line segment. **(G,H)** The MR has disappeared. The blue line segments indicate the MV annular plane.

The distribution of patients undergoing mitral valve repair according to these subtypes is shown in Figure 4A. All patients undergoing concurrent mitral valve repair achieved immediate postoperative resolution of mitral regurgitation or no regurgitation, with significant improvement in morphology, increased coaptation length, and increased coaptation depth (Figure 5).

### Postoperative follow-up of cardiac chamber size and mitral valve regurgitation changes

In the observation group, the diameters of the right atrium and right ventricle were significantly reduced at discharge (within postoperative 7 days) compared to preoperative measurements, and the differences were statistically significant (*P* < 0.05). No further significant reduction was observed at postoperative 6 or 12 months.

The left ventricular diastolic diameter (LVEDD), left ventricular systolic diameter (LVESD), left ventricular diastolic volume (LVEDV), and left ventricular systolic volume (LVESV) increased at postoperative 6 months compared to preoperative and discharge measurements, and the differences were statistically significant (all *P* < 0.05). No further significant increase was observed at postoperative 12 months.

The maximum flow velocity across the MV at discharge increased compared to preoperative levels, and no further increase was observed at postoperative 6 or 12 months.

At postoperative 3 months, 3 patients developed moderate mitral regurgitation (since no preoperative mitral regurgitation was present, mitral valve repair was not performed concomitantly). Among these 3 patients, 2 were classified as ASD-MV type II and 1 as type Ib. Follow-up continued for 2 years, during which 2 cases progressed to moderate regurgitation and 1 to severe regurgitation. Details are shown in Table 4.

**TABLE 4:** Transthoracic echocardiographic observation of preoperative and postoperative changes in cardiac chambers and mitral valve-related parameters ($\bar{\text{x}}$ ± s).

Project	LVEDD (mm)	LVESD (mm)	LVEDV (mL)	LVESV (mL)	LAESD (mm)	RVEDD (mL)	RAESD (mL)	LVEF (%)	MV-Vmax (cm/s)	MR>Moderate (n)
Pre	42.5 ± 3.8	29.2 ± 3.4	67.3 ± 14.6	27.4 ± 7.5	35.4 ± 5.2	42.4 ± 7.5	49.9 ± 9.0	58.6 ± 4.6	70.3 ± 31.5	7
Post										
1 week	42.6 ± 3.5	29.5 ± 2.9	68.1 ± 14.5	28.0 ± 6.8	34.7 ± 5.3	27.4 ± 5.1*	35.3 ± 6.9*	58.3 ± 3.3	91.3 ± 28.6*	0
6 month	44.9 ± 3.3^*^^#^	30.7 ± 3.1^*^^#^	77.2 ± 15.4^*^^#^	31.3 ± 7.7^*^^#^	35.8 ± 5.1	26.3 ± 4.4*	36.1 ± 6.5*	59.3 ± 3.5	93.8 ± 29.1*	3
12 month	45.0 ± 3.2^*^^#^	30.9 ± 3.1^*^^#^	78.4 ± 14.2^*^^#^	31.9 ± 7.8^*^^#^	35.4 ± 4.8	26.5 ± 4.0*	35.8 ± 6.9*	59.1 ± 3.9	94.2 ± 30.6*	3
*F*	12.529	5.458	10.941	6.481	0.710	157.119	68.775	0.741	13.516	–
*P*	<0.001	0.001	<0.001	<0.001	0.546	<0.001	<0.001	0.528	<0.001	–

LVEDD, left ventricular end-diastolic diameter; LVESD, left ventricular end-systolic diameter; LV-EDV, left ventricular end-diastolic volume; LV-ESV, left ventricular end-systolic volume; LAESD, left atrial end-systolic diameter; RVEDD, right ventricular end-diastolic diameter; RAESD, right atrial end-systolic diameter; LVEF, left ventricular ejection fraction. MV-Vmax, maximum transmittal mitral valve flow velocity;

*P < 0.05 compared with preoperative data;

^#^*P* < 0.05 compared with 1 week after operation. 1 mmHg = 0.133 kPa.

Observations indicated that three patients with newly developed moderate-to-severe mitral regurgitation had a Qp/Qs ratio ≥ 2.8. specifically measuring 4.1, 4.3, and 3.8 respectively. After the operation, the anteroposterior diameter of the left ventricle during diastole increased significantly. One case’s diameter expanded from 37 to 49, 44 to 50 mm, and from 39 to 48 mm in the three postoperative ultrasound examinations. The anteroposterior diameter of the mitral valve annulus also showed a significant increase compared to the preoperative state.

These findings suggest that the occurrence or progression of mitral regurgitation is not only associated with the morphology of the mitral valve and the preoperative regurgitation status while also correlated with an excessively high Qp/Qs ratio before ASD repair. The literature shows that the Qp/Qs ratio is strongly correlated with left ventricular enlargement after ASD repair, and a Qp/Qs ratio ≥ 2.8 is an independent risk factor (16). The results demonstrated that 34 (45.3%) patients in the observation group had a Qp/Qs ratio ≥ 2.8, and over 50% of patients in types Ib, II, and III exhibited this ratio (Figure 4B). Higher Qp/Qs values are associated with a greater likelihood of mitral valve morphological abnormalities and an increased risk of postoperative regurgitation exacerbation.

## Discussion

Studies have shown that 29–35% of patients experience worsening or new-onset MR after ASD repair (17, 18). Following ASD repair, early-stage MR may occur due to the increased volume load on the left ventricle, which causes the ventricular septum to shift toward the right side of the heart (17). The pathogenesis of long-term MR exacerbation after ASD repair remains incompletely understood. However, 87.5% of patients with significant MR progression exhibit mitral annular dilation, compared to only 6.1% of those with no or mild progression. Therefore, mitral annular dilation may play a critical role in the medium to longterm progression of two or more regurgitation grades (19). Some scholars suggest that after ASD repair, the increased left ventricular end-diastolic volume may lead to left ventricular or mitral annular dilation, thereby contributing to MR exacerbation. Consequently, some patients require concurrent mitral valve repair during ASD surgery (20). In this study, the mean left ventricular anteroposterior diameter and end-diastolic volume (EDV) in the observation group increased at postoperative 6 months compared to preoperative values. Among the 3 patients with moderate to severe postoperative regurgitation, significant left ventricular anteroposterior diameter enlargement was observed, accompanied by mitral annular dilation.

Further research is warranted to determine under what specific circumstances ASD patients require concurrent mitral valve repair.

Clinical practice has revealed that patients with increased MR after ASD repair often exhibit morphological abnormalities of the mitral valve. Studies suggest that long - term ASD leads to right ventricular dilation, causing the leftward displacement of the ventricular septum. This, in turn, results in the inward displacement of the left ventricular papillary muscles and a shortening of the intermuscular distance, potentially leading to relatively elongated chordae tendineae. This may be one of the mechanisms underlying the morphological abnormalities of the mitral valve leaflets (16, 21). This study proposes, for the first time, a classification of ASD patients into four types based on the degree of morphological abnormalities of the mitral valve, termed the ASD-MV classification. Among them, types II and III (accounting for 37.3% of the observation group) are characterized by leaflets protruding beyond the mitral annulus plane and prolapsing into the left atrium, accompanied by significant morphological abnormalities.

This study involved 33 patients undergoing simultaneous mitral valve repair. Among them, 26 (34.7% of the total observation group) were classified as ASD-MV types II and III with preoperative mild to moderate or severe MR. Seven cases included those with moderate or above MR before the operation or those with moderate or above MR immediately after ASD repair. All patients in the observation group were followed up for 1 year after surgery. Three cases (4.0%) exhibited an increase in MR to moderate or severe levels, which was significantly lower than the reported incidence in the literature. This suggests the feasibility of the proposed intervention method.

Among the three patients with increased MR to moderate or higher levels after surgery (since no MR was present so no mitral valve intervention was performed), two patients were classified as ASD-MV type II and one as type Ib. The underlying mechanism may involve a large ASD preoperatively, which caused significant left to right shunting at the atrial level, resulting in an excessively high Qp/Qs ratio. After surgery, the left to right shunting at the atrial level was resolved, leading to an increase in left ventricular blood volume and subsequent enlargement of the left ventricle and mitral annulus. However, these patients presented with mitral valve morphological abnormalities, featuring insufficient leaflet coaptation length and coaptation depth, making it difficult to tolerate the effects of left ventricular and mitral annular enlargement following ASD repair. Consequently, significant MR was observed.

Other studies indicate that a Qp/Qs ratio ≥ 2.8 may suggest significant postoperative right ventricular septal displacement and serves as an independent risk factor for new or aggravated mitral regurgitation following ASD repair (6). Therefore, when considering concurrent mitral valve repair, in addition to evaluating mitral valve morphological abnormalities, attention should also be paid to the Qp/Qs ratio. In this study, three patients developed moderate regurgitation immediately after ASD repair, including one with type Ia and two with type Ib. All three patients had a Qp/Qs ratio ≥ 2.8.

The surgical approaches for mitral valve repair vary according to ASD-MV subtypes as follows. Type I patients only need artificial annuloplasty to reduce the annulus. For Type II and III patients, the tip of the anterior leaflet must be repositioned to a more normal position. When the tip of the posterior leaflet protrudes beyond the annular plane, artificial chordae tendineae should be used for restoration. If the leaflet tip is aligned with the annular plane, generally no intervention is required, because the tip of the posterior leaflet often shifts toward the left ventricle due to the original chordae tendineae after annuloplasty, resulting in a position below the annular plane.

In the observation group, patients who underwent concurrent mitral valve repair showed no or minimal postoperative regurgitation with significant morphological improvement. Unlike the traditional Carpentier classification system, the classification method employed in this study focuses on morphological ultrasonic imaging of valves, offering a more intuitive and visual approach. Furthermore, this classification system provides a more detailed categorization of valve lesions similar to those classified as Type II by Carpentier, thereby providing more comprehensive surgical guidance for patients with atrial septal defects. As all patients enrolled in the present study received surgical ASD repair. Therefore, our findings are primarily based on data from patients treated with surgery. The ASD in patients who underwent this surgical approach was relatively large in area, and the location or size of the defects did not meet the indications for transcatheter closure. Whether the classification method proposed in this paper is applicable to patients receiving transcatheter closure remains to be verified with accumulated clinical cases. Based on our previous clinical experience, larger atrial septal defects are more likely to be complicated by mitral valve morphological abnormalities (such as ASD-MV type II or type III). When evaluating whether a relatively large ASD is suitable for transcatheter closure, our classification system carries important guiding significance.

## Limitations

First, elderly patients with atrial septal defect are often complicated with atrial fibrillation, which is also one of the critical risk factors exacerbating mitral regurgitation (2, 6, 9, 22). To reduce confounding bias in this study, patients with preoperative arrhythmias were excluded. Nevertheless, the significant clinical correlation of this factor cannot be ignored, and further research with larger sample sizes is required for subsequent exploration. Second, standardized quantitative reproducibility analysis has not been performed for this novel ASD-MV classification system. Although the article mentions that all echocardiographic image analyses were independently completed by two experienced echocardiographers with acceptable analytical consistency, inter-observer consistency indicators such as Cohen’s Kappa coefficient and intraclass correlation coefficient were not calculated for standardized verification. This study is a single-center retrospective research without a pre-designed reproducibility sub-study, so the reliability of this new classification criterion remains unconfirmed. To widely apply this classification standard to routine clinical diagnosis and treatment, prospective multicenter external validation should be conducted, accompanied by quantitative assessment of inter-observer and intra-observer variability. Thirdly, the Qp/Qs values were not measured using right heart catheterization combined with the Fick method (gold standard), instead, they were obtained via TEE (which has certain reference value). Furthermore, the follow-up period in this study was only 1 year, which is relatively short. Therefore, we cannot fully evaluate the long-term stability of mitral valve repair and late recurrence of mitral regurgitation after ASD correction. Extended follow-up is required to confirm long-term outcomes. Last but not the least, patients with dizziness or suspected PFO may have subtle underlying constitutional or hemodynamic differences compared with the general healthy population, which could influence mitral valve morphology. The control group comprised patients undergoing TEE for evaluation of dizziness or suspected PFO rather than fully healthy individuals. TEE is an invasive examination and is not performed in asymptomatic healthy subjects under routine clinical care, limiting the availability of an ideal healthy control cohort. Selection bias cannot be excluded: patients evaluated for dizziness/PFO may have subtle physiological characteristics different from the general population, which might affect mitral valve morphology. Accordingly, the between-group comparisons should be interpreted with caution.

## Conclusion

This study found that adult patients with a large ASD often have structural abnormalities of the mitral valve. Our previous clinical experience at our hospital has shown that such abnormalities raise the risk of postoperative mitral regurgitation. This study suggests that for adult patients with type I ASD, especially those with a pulmonary-to-systemic blood flow ratio (Qp/Qs) ≥ 2.8, close monitoring of changes in the severity of MR is required after weaning from cardiopulmonary bypass during ASD repair. If a marked increase in regurgitation is detected intraoperatively, concomitant mitral valvuloplasty is recommended. For patients with type II or type III ASD-MV classification ASD and a preoperative Qp/Qs ≥ 2.8, concomitant mitral valvuloplasty during ASD repair is more appropriate even if no MR is present preoperatively. The 1-year postoperative follow-up of this study recorded three cases (4%) of new-onset or significantly aggravated MR, a figure substantially lower than rates reported in prior literature.

Notably, the data obtained in this study only yield preliminary exploratory conclusions. Constrained by a small total sample size, limited case numbers in each subgroup, and few cases of progressive postoperative MR, we failed to identify independent risk factors that can effectively predict adverse mitral valve outcomes. Larger-scale prospective studies are still needed to verify the aforementioned preliminary surgical diagnosis and treatment recommendations. The surgical indications and reference criteria for concomitant mitral valvuloplasty proposed in this paper should be adopted with caution in clinical practice. The present 1-year follow-up data demonstrate favorable early and mid-term outcomes of the proposed surgical strategy. Further long-term observation is needed to evaluate the durability of mitral repair and late recurrence of MR.

## Data Availability

The original contributions presented in this study are included in the article/supplementary material, further inquiries can be directed to the corresponding authors.
